# Design and Analysis of Bionic Cutting Blades Using Finite Element Method

**DOI:** 10.1155/2015/471347

**Published:** 2015-12-07

**Authors:** Mo Li, Yuwang Yang, Li Guo, Donghui Chen, Hongliang Sun, Jin Tong

**Affiliations:** ^1^The Key Laboratory of Bionic Engineering, Ministry of Education, Jilin University, 5988 Renmin Street, Changchun 130022, China; ^2^The School of Mechanical Engineering, Xi'an Jiaotong University, Xianning Road, Xi'an 710049, China

## Abstract

Praying mantis is one of the most efficient predators in insect world, which has a pair of powerful tools, two sharp and strong forelegs. Its femur and tibia are both armed with a double row of strong spines along their posterior edges which can firmly grasp the prey, when the femur and tibia fold on each other in capturing. These spines are so sharp that they can easily and quickly cut into the prey. The geometrical characteristic of the praying mantis's foreleg, especially its tibia, has important reference value for the design of agricultural soil-cutting tools. Learning from the profile and arrangement of these spines, cutting blades with tooth profile were designed in this work. Two different sizes of tooth structure and arrangement were utilized in the design on the cutting edge. A conventional smooth-edge blade was used to compare with the bionic serrate-edge blades. To compare the working efficiency of conventional blade and bionic blades, 3D finite element simulation analysis and experimental measurement were operated in present work. Both the simulation and experimental results indicated that the bionic serrate-edge blades showed better performance in cutting efficiency.

## 1. Introduction

The performance of cutting tool mainly depends on the geometry of cutting blade, soil mechanical properties, soil texture, and the operating conditions (such as forward speed and cutting depth) [1, 2]. Theoretical analysis and experimental methods should be utilized for achieving adequate design of the efficient soil-cutting tools for breaking soil and digging out stubbles from field. These cutting tools should be energy-efficient and able to cut soil and lead to a suitable soil condition for crop growth next year. Generally, the performance tests and field tests in tillage tool design and development are time-consuming and costly. Accurate modeling of soil-implement interaction is the basic key to the optimization of tillage tool design and may omit the need for numerous expensive field tests and shorten the research time [1]. Finite element method (FEM) is a powerful numerical technique and nonlinear behavior of tillage tool interaction with soil can be modeled if a proper constitutive law is chosen in finite element analysis (FEA). Besides, FEM is good at analyzing complex engineering problems, especially for dynamic systems with large deformation and failure [3, 4]. It has been used by many researchers [5–9] to analyze problems related to soil mechanics and the interaction between soil and tillage tools.

Bionic method can transfer biological solutions to engineering techniques and it has been developed as an effective application of biological principles and methods to engineering [10]. Some researchers found that bionic design could improve working efficiency of blades. Ji et al. designed a rotary blade by mimicking the front claw toe of mole [11]. Ren et al. made an experimental investigation of soil-burrowing animals' body surfaces and designed some bionic curved soil-cutting blades to reduce soil adhesion and friction [12]. Meyers et al. proposed a scissor design based on the ability of piranha teeth to cut through flesh [13]. As an efficient predator in the insect world, the praying mantis has a pair of powerful tools, two sharp and strong forelegs. The femur and tibia of the foreleg are both armed with a double row of strong spines along their posterior edges, which can firmly grasp the prey, when the femur and tibia fold on each other in capturing [14]. Moreover, the tibia can cut into the prey body quickly and efficiently, since the sharp and hard tibia spines have the same effect of sawteeth, which can increase the contact pressure and decrease the power. It is conceivable that the profile and shape of these tibia spines will be beneficial for the design of bionic cutting tools.

The objective of this work is to mimic the geometry of tibia spines on praying mantis (*Mantis religiosa* Linnaeus) to design a new kind of stubble cutting tool. The FEA approach was employed to compare cutting force of bionic blades with a conventional smooth-edge blade. Soil was modeled as an elastic-plastic material that revealed material hardening, and the linear form of the extended Drucker-Prager yield criterion was used. The soil mechanical property parameters were obtained from triaxial tests. Simultaneously, specialized laboratory equipment was designed and built to measure the cutting force of these blades.

## 2. Materials and Methods

### 2.1. Geometrical Characteristics of Praying Mantis's Foreleg

**Figure 1:**
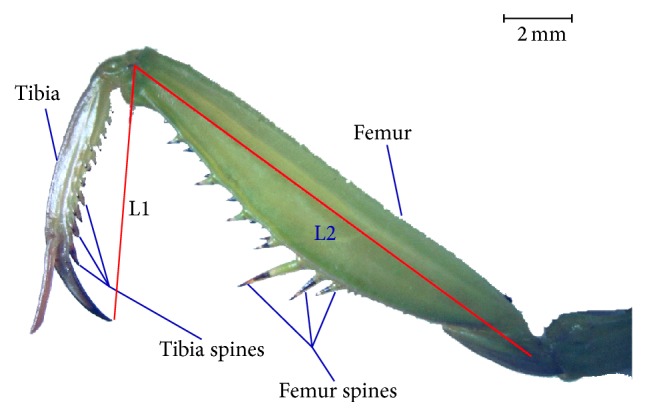
Praying mantis's foreleg.

The praying mantises used in this work, which were found in the countryside of China Northeast, belong to the species of* Mantis religiosa* Linnaeus. As for morphology measurement, 25 adult praying mantises were cleaned by distilled water and then anesthetized with ether. During the anesthesia phase, these praying mantises were observed and photographed by microscope (STJ-30, Olympus Co., Ltd.). The sharp and strong forelegs are armed with a double row of strong spines along their posterior edges, as shown in Figure 1. The length range of these mantises is 45~65 mm, and the total length of tibia and femur is from 16.5 mm to 24.7 mm. Tibia spines are of different size and have a close arrangement, and the height of spines is about 10~30% of the width of tibia, which make it look like a sharp saw.

### 2.2. Design of Bionic Cutting Blade

Traditionally, cutting equipment of harvesting machinery for rhizome crops is designed based on the working principle of wedge [15]. Considering that wedge-shape tool has simple geometry structure and is easily manufactured, a series of cutting blades were designed in this work. The conventional blade firstly cut down the stubble, and the complex of soil and stubble would move along the blade face and then fell on the ground. The conventional smooth-edge blade can not only cut down the stubbles but also break the ridge; it plays an important role in the soil preparation work.

**Figure 2:**
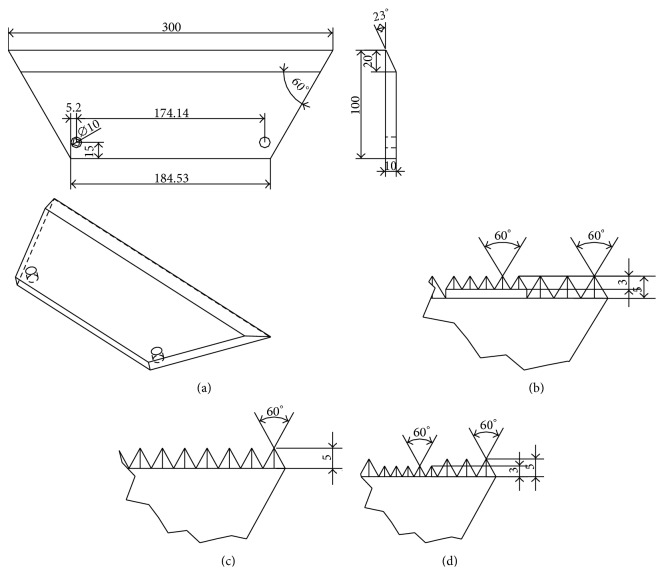
(a) Reference blade A, (b) bionic blade B, (c) bionic blade C, and (d) bionic blade D (mm).

According to the research of praying mantis' foreleg, the geometrical structure of tibia plays a significant role in its prey function. The sharp and hard tibia spines have the same effect of sawteeth, which can increase the contact pressure and decrease the leg power. Thus, the tibia can cut into the prey body quickly and efficiently. The geometrical characteristic of praying mantis' tibia can be adopted in the design of cutting blade, since the cutting process has a similar way as the cutting of tibia. A conventional smooth-edge blade with no bionic geometrical element was used as a reference blade, shown in Figure 2(a). Learning from the arrangement of tibia spines, two different sizes of sawteeth geometrical elements were designed on the cutting edge of conventional blade. According to measurement of spines height, it was found that the spine height range of 10%–30% of tibia width was appropriate for cutting. Then, some blades with different teeth height (3 mm, 5 mm, 7 mm, and 10 mm) were modeled in simulation software in the early parameter research, and it proved that blades with teeth height of 3 mm and 5 mm were representative in resistance reduction. Figure 2(a) shows the conventional blade with flat cutting edge, and Figures 2(b), 2(c), and 2(d) show the geometrical structure of bionic serrate-edge blades. There are two sizes of tooth shape rectangular pyramid on the cutting edge of blade B, the height of big tooth is 5 mm, and the small tooth is 3 mm. The big teeth and small teeth were added on the cutting edge in an interlaced pattern, and the envelope line of teeth top is still a straight line. Blade C, different from blade B, only has the small cutting teeth on its cutting edge. Similar to blade B, blade D has an alternate arrangement of big and small teeth; however, the envelope line of teeth top is not a straight line.

### 2.3. FE Model Description

In this work, the finite element simulation was performed in ABAQUS to analyze and compare the working performance of conventional blade and bionic blades.

#### 2.3.1. Soil Model

Soil is a complex material consisting of three phases, namely, solid, liquid, and gaseous phases, within which a number of different physical, biological, and chemical processes control soil mechanical behavior [16]. Under external loading, soil will show elastic-plastic behavior with a geometrical nonlinearity [2], and plasticity theory applicable to soil mechanics was well reviewed in literature [17, 18]. Moreover, many yield criteria have been put forward for soil constitutive models that are defined generally when maximum stress state or maximum strain energy reaches a critical value. The Mohr-Coulomb, Cam-clay, and Drucker-Prager yield criteria have shown significant applications in soil mechanics.

In this work, agricultural soil was recognized as an elastic-plastic material with Drucker-Prager criterion. The yield function of the Drucker-Prager criterion could be described as [19]

(1) $$\begin{array}{c} f=3\alpha \sigma_{m}+\overset{-}{\sigma }-k=0, \end{array}$$

where *α*, *k* are material parameters; *σ*_*m*_ is mean compressive stress that can be written as the first-stress invariant, *I*_1_:

(2) $$\begin{array}{c} \sigma_{m}=\frac{1}{3}I_{1}=\frac{1}{3}\left(\;\sigma_{x}+\sigma_{y}+\sigma_{z}\;\right), \end{array}$$

where $\bar{\sigma }$ is effective stress, and it is could be related to the second-derivative stress invariant, *J*_2_:

(3) σ¯=J2,J2=12σx−σm2+σy−σm2+σz−σm2+τxy2+τyz2+τxz2,

where *τ* is shear stress and *σ* is compressive stress.

From (3), Drucker-Prager model could account for both soil volumetric and shear behaviors.

**Figure 3:**
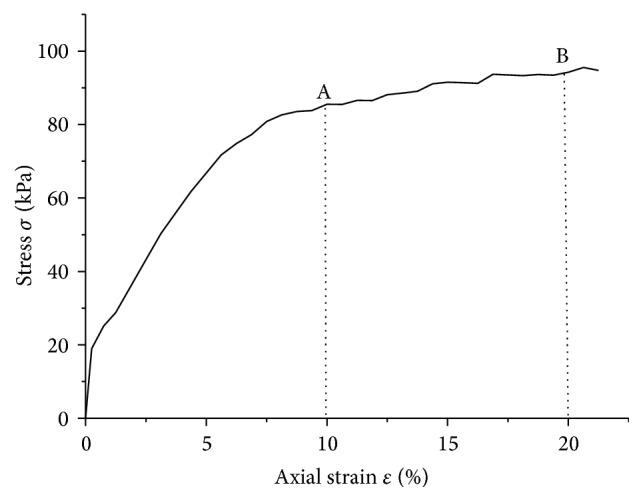
Stress-strain curve of the sample soil: A is theoretically considered as the yield point; B is theoretically considered as the failure point.

In order to authentically simulate the cutting force, the sample soil was taken from the countryside field. In triaxial compression test, four soil samples were used in the laboratory triaxial compression tests, and the specimen shape was cylinder with a size of Φ39.1 cm × 80 cm. The shear strain rate was 0.4 mm/min and the confining pressure was loaded by 100 kPa, 200 kPa, and 300 kPa, successively. The parameters required in this finite element model are bulk density (*ρ*); Young's modulus (*E*); Poisson's ratio (*ν*); the angle of friction (*β*); yield stress ratio of triaxial tension to triaxial compression (*k*); dilation angle (*ψ*) for the plastic flow and soil-metal friction coefficient. Each of these values is the average of 5 field samples and all the data are shown in Table 1. Modeling the hardening behavior is of great significance in agricultural soil, since soil would experience compression under loading before it fails. The hardening data was related to the stress-strain curve (Figure 3) acquired from the triaxial compression test. The equivalent plastic strain which is required in the computer model can be expressed as [20]

(4) $$\begin{array}{c} \varepsilon^{pl}=\varepsilon^{t}-\frac{\sigma }{E}, \end{array}$$

where *ε* ^*t*^ is total strain (elastic strain plus plastic strain) and *σ* is yield stress.

#### 2.3.2. Model Dimensions and FE Mesh

**Figure 4:**
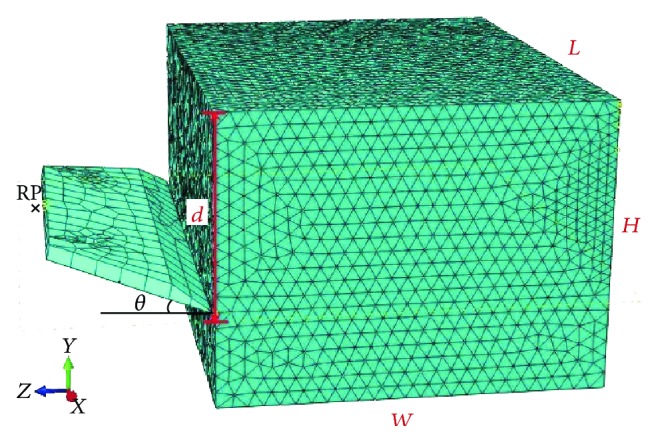
Soil-cutting model dimensions.

The 3D model dimensions are presented in Figure 4 and Table 2, where “*L*” is the length of the soil body, “*W*” is the width, “*H*” is the height, “*d*” is the cutting depth, and “*θ*” is the wedge angle. The blade was assumed as rigid body with the reference point (RP), which is located in the middle location of blade rear. Simulating the blade as a “rigid body” in this work could make sure that the calculation of reaction force working on the entire blade converges at one single reference point.

In FEA, the materials of blades were defined as steel C45. The blades were divided into three-dimensional 8-node linear brick continuum elements (C3D8R) and the soil was divided into 10-node modified quadratic tetrahedron elements (C3D10M). The numbers of nodes and elements for soil body were 143650 and 102011, respectively.

#### 2.3.3. Boundary and Load Conditions

In order to appropriately model the dynamic interaction of soil and cutting blade, various boundary and loading conditions were applied.

The bottom of the soil part was fully constrained in all six translational and rotational degrees of freedom. Other surfaces of the soil were not constrained. The rigid blade was constrained with a constant translational velocity. A forward velocity of 500 mm/s was used in this model, different from that of experiments (the difference between the velocities in the simulation and the tests was explained in Section 3.2). Gravity effect was taken into consideration by applying the gravity acceleration as a load in all steps. General interaction feature was used in explicit module to describe the interaction relationship of blade and soil. Interactions between the contact surfaces consist of two parts: tangential behavior and normal behavior. The parameter of tangential behavior used in this model is a friction coefficient between blade and soil. This coefficient was measured using soil adhesion measurement apparatus, referenced from early research paper, and the coefficient was chosen as 0.42 [7]. A commonly used “hard” contact was adopt to describe the normal behavior.

### 2.4. Experimental Measurement

**Figure 5:**
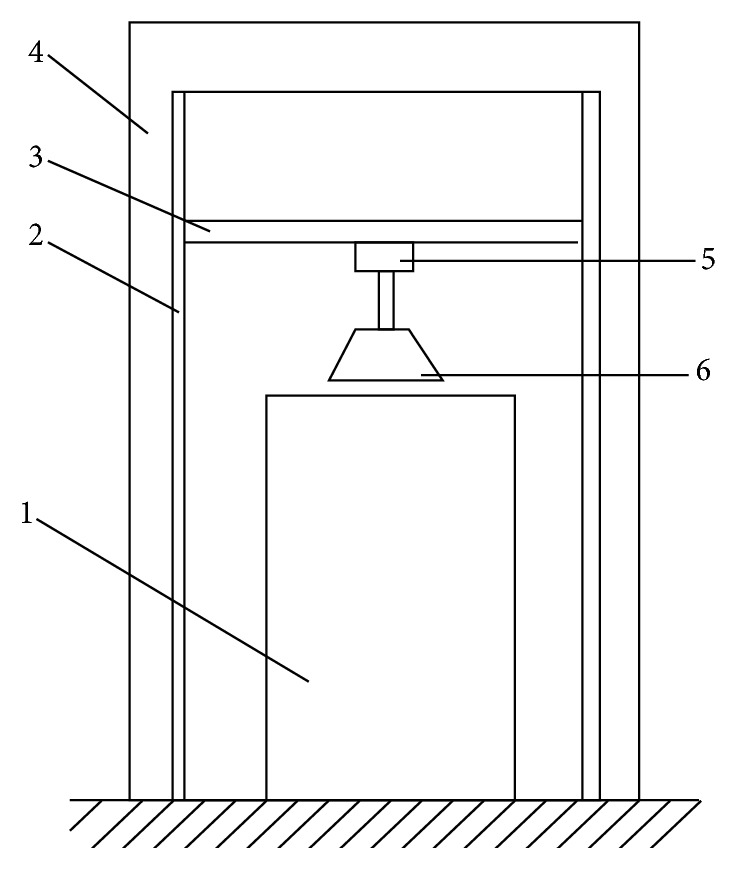
A schematic diagram of the testing device.

In order to examine the influence of cutting edge shape on soil-cutting force, a reformed experimental device was used to execute the soil-cutting tests. A schematic diagram of the testing device is presented in Figure 5. The whole device was supported by a steel frame (4). A soil bin (1) of 1 m height and 0.5 m × 0.5 m length and width was placed below the blade (6). A beam (3) could move up and down along a slideway (2). By applying a fixed speed to make the blade move down and cut into the soil, a force sensor (5) mounted on the beam will online record the test curve of force versus time and force versus displacement. The velocity range of movable beam is between 1 mm/s and 8.5 mm/s. Three vertical velocities (*V*_1_ = 2 mm/s, *V*_2_ = 6 mm/s, and *V*_3_ = 8.5 mm/s) and a constant soil-cutting displacement (50 mm) were adopted in this work.

The experimental soil in the soil bin was taken from the farming field, same as that in simulation model. Based on the standard for soil test method (Chinese Standard for soil test method, GB/T50123-1999), the farming soil is typical fine-grained loam, and the soil particle distribution is listed on Table 3. The samples were taken from three replicate plots of field area and each plot was 10 × 5 m. All soil samples came from the cultivated layer and from a depth of 100 mm. Hardness of the field soil was measured with a cone penetrometer, and the average cone index value was 0.55 MPa. Each blade had 5 repeated tests, each test started after placing the blade at required height, and the soil would be flipped and pressed to the hardness of 0.55 MPa.

## 3. Results and Discussion

### 3.1. Simulation Results

Table 4 describes the simulation results of the initial and final soil-cutting moment for those blades. The blades gradually cut into soil and made some soil elements distorted with time increasing. It can be seen that the magnitude of soil stress in model of blade A at initial cutting moment is slightly higher than the bionic blades, and the soil stress at final moment approximated to bionic ones. The changing soil stress indicates the blades gradually cause some soil elements to be deformed and fail. Thus, the four blades bore similar resistance force in the whole cutting process.

**Figure 6:**
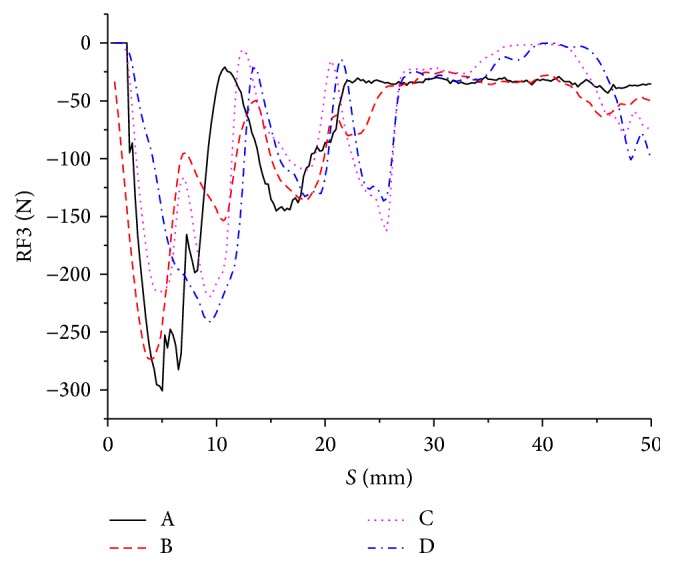
Reaction force of 4 blades along the *z*-axis.

In this work, reaction force at the reference point (RP) can delegate the cutting force of whole blade. The reaction force of RP on *z*-axis direction (RF3) is the focus of this research, shown in Figure 6. It can be found that RF3 increases rapidly at first (0–5 mm); then it gradually decreases and has some fluctuation in the initial cutting stage (5–25 mm). After 20 mm, the cutting process tends to be smooth and steady; thus all the RF3 curves rise gradually and then decrease before a level off. The difference of cutting process between bionic serrate-edge blades and conventional smooth-edge blade without bionic structure is that the contact area of cutting edge of bionic blades on soil is smaller than that of smooth-edge blade A. So the resistance force of bionic blades would be lower than blade A during the initial stage of cutting process. Moreover, RF3 of blades B, C, and D is higher than blade A at first (0–7 mm). After the tooth surface of bionic blades completely cuts into soil and moves forward smoothly, soil in front of the teeth gradually fails and reaction force keeps falling and then levels off. The reaction force of blade A is similar to the bionic ones while the cutting edge is almost completely cut into soil (10–25 mm). What is more, the area under RF curve shows the work of the applied force needed for the blade cutting into soil and moving through it. According to integral calculation, the cutting work of blade C shows lowest value of 3480 mJ, which is lower than that of conventional blade A by 4.3% and then blade D (3590 mJ). Blade B has the highest work value among all these blades. The explanation for the larger cutting work of blade B could be that the platform beneath small teeth had a certain thickness which would increase the contact area between cutting edge and soil, and the resistance force rises as well.

### 3.2. Experiment Results

**Figure 7:**
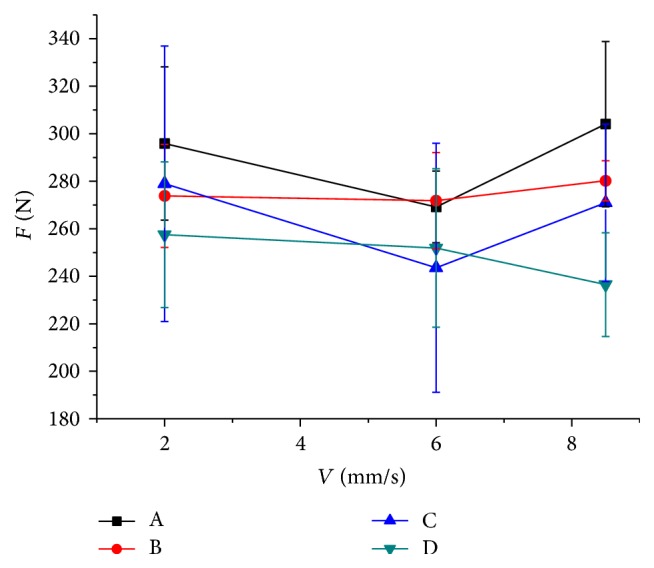
Experimental results of the maximum soil-cutting resistance.

Figure 7 demonstrates the maximum cutting force of 4 blades on varied velocities, in which the error bars show the corresponding standard deviation. It can be found that the influence of cutting speeds on cutting force is not significant. However, different cutting edge shapes had obvious influence on soil-cutting force. The soil texture is not uniform since it was taken from natural field, which leads to some experimental errors. However, the cutting force of blade A was much higher than other blades with bionically toothed structure in most cases. Blades D and C have similar force and blade D shows a relatively lower value. To some extent, the measurement result is consistent with simulation result. Besides, an *F* test was adopted to do the significance test, and the results of *F* tests for correlation between the blade type and cutting speed of the cutting force are summarized in Table 5. A significant value lower than 0.05 indicates the blade type has significant effect on cutting force. On the contrary, the cutting speed has no significant effect on it. This result is consistent with the previous researches [1]. Actually, Bekker proclaimed that the speed in sinkage tests is inappreciable until the rate is up to 740 mm/s [21].

**Figure 8:**
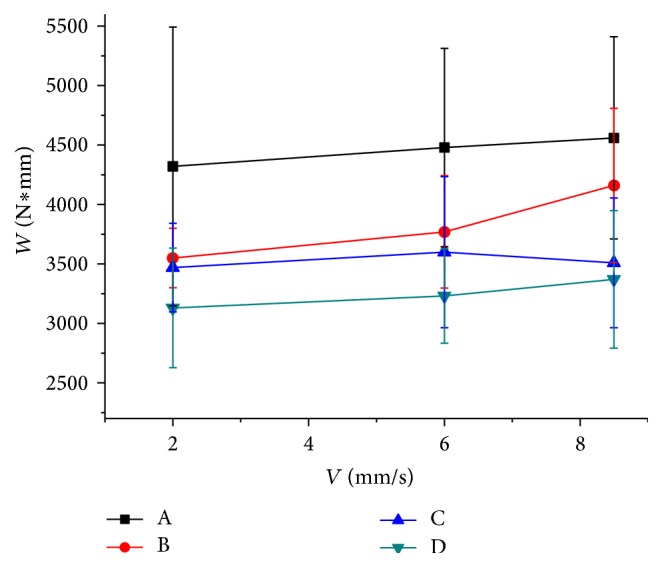
Soil-cutting work of 4 blades.

During the soil-cutting process, the blade cut into soil at uniform velocity, and vertical force rose with increase of displacement. Thus, the force versus displacement curve can be recorded, and area under the curve represents the work of applied vertical force required for the blade traveling a certain distance (50 mm). The cutting works of 4 blades were calculated and compared (Figure 8). Similar to the force measurement results, cutting speed had no obvious effect on the work. The highest cutting work was that of blade A and the lowest work was that of blade D. Blade B has a larger value among bionic blades and it is higher than blade A in some cases. The results of *F* tests also showed the blade type had significant effect on the work and the cutting speed did not (Table 6).

In our early research, a bionic rotary disc was designed by learning geometrical structure of the mole rat's claw toes [22]. Though simulation analysis showed that the bionic disc performed better in structure strength and soil-cutting function than a conventional disc, the rotary cutting of bionic disc would depend on the tractor power and consume a lot of energy. However, the working way of the bionic sawteeth blades designed in present work is horizontal cutting with tractor forward speed, which can greatly reduce energy.

## 4. Conclusions

This work examined the geometrical structure of praying mantis's foreleg, and the characteristic geometry structure of tibia was utilized in reformed design of a stubble cutting blade. Learning the arrangement of tibia spines, two different sizes of sawteeth geometrical elements were designed and added on the cutting edge of conventional blade. Based on the simulation and experimental results, it can be concluded that the bionic serrate-edge blades had better performance than conventional smooth-edge blade in improving the soil-cutting efficiency. From the FE analyses and actual measurement, some remarks can be obtained as follows:

(1) The 3D dynamic FE model analysis showed the magnitudes of soil stress in bionic blade cutting models are lower than that in conventional blade. RF3 of blades B, C, and D is smaller than blade A at first, and the reaction force of blade A is similar to the bionic ones while the cutting edge is entirely into the soil. The cutting work of bionic blades C and D is lower than that of blade A.
(2) The experimental tests indicated the maximum cutting force of blade A was much higher than the bionic ones. Blade C and blade D showed lower cutting force and work.
(3) *F* tests indicated the blade type has significant effect on cutting force and cutting work, but the cutting speed did not.

## Figures and Tables

**Table 1:** Soil property parameters required in the FEM model.

Bulk density, *ρ* (Mg/m^3^)	Young's modulus, *E* (MPa)	Poisson's ratio, *ν*	Friction angle, *β*	Stress ratio, *k*	Dilation angle, *ψ*	Soil moisture, %
1.79	1.14	0.3	12.21°	1	0°	20.03

**Table 2:** Dimensions of the three-dimensional soil-cutting model.

*L* (mm)	*W* (mm)	*H* (mm)	*d* (mm)	*θ* (deg)
260	200	150	100	12

**Table 3:** Particle distribution of testing soil.

Particle size (*μ*m)	<4	4~30	30~75	75~250	>250

Content (%)	1.56	27.31	12.88	58.25	0

**Table 4:** Simulation result.

Blade	Maximal values of the von Mises stresses (MPa)	
	Initial cutting moment	Final cutting moment
A	0.2102	0.2506
B	0.3003	0.2531
C	0.2625	0.2255
D	0.2649	0.2663

**Table 5:** *F* test results—dependent variable: cutting force.

	*F*	Sig.
Cutting speed	0.255	0.783
Blade type	17.306	0.002

**Table 6:** *F* test results—dependent variable: work.

	*F*	Sig.
Cutting speed	3.661	0.091
Blade type	40.949	0.000
